# Supplementary material for: Truncated Expression of a Carboxypeptidase A from Bovine Improves Its Enzymatic Properties and Detoxification Efficiency of Ochratoxin A
Source: Toxins (Basel). 2020 Oct 29;12(11):680. doi: 10.3390/toxins12110680 (PMC7692142; doi:10.3390/toxins12110680)
Supplement: Supplementary file 1 [file toxins-12-00680-s001.pdf]

Supplementary Materials: Truncated Expression of a Carboxypeptidase A from Bovine Improves Its Enzymatic Properties and Detoxification Efficiency of Ochratoxin A

Lu Xiong Mengxue Peng, Meng Zhao and Zhihong Liang

1. M-CPA effectively degraded OTA in vitro

Methods: The in vitro gastrointestinal model simulating the digestive processes of the stomach and small intestine was used as the methods described by Jiang et al (2010). Simulated gastric juice: 0.8 g of pepsin and 4.375 g of NaCl, dissolved in 250 mL of distilled water, adjusted to pH 1.2 with hydrochloric acid. Simulated intestinal juice: 0.143 g trypsin and 0.857 g bile, dissolved in 100 mL of 0.1 mol/L $NaHCO_3$ solution with pH 6.0.

For the gastric stage, 1.8 mL of gastric juice was added to the tube sets containing OTA (2 μg/mL) and M-CPA (10 U/mL)/S-CPA (10 U/mL), and the mixtures were rotated for 3 h and then collected 0.5 mL mixture every 1 h for further survival OTA extraction.

In the intestinal digestion stage, 1.8 mL of intestinal juice was added to the remaining tubes and incubated with rotation for another 3 h, which was then taken out for further OTA and OTα analysis every one hour.

1.1 The reference of method:

Jiang, L., Olesen, I., Andersen, T., Fang, W. & Jespersen, L. (2010) Survival of Listeria monocytogenes in Simulated Gastrointestinal System and Transcriptional Profiling of Stress- and Adhesion-Related Genes, Foodborne Pathogens And Disease. 7, 267-274.

1.2. Results

An *in vitro* simulated gastrointestinal digestion model was used to evaluate whether M-CPA can stably degrade OTA in the gastrointestinal digestive environment. M-CPA and S-CPA were incubated with OTA in a simulated gastrointestinal digestion model for 3 h, and TLC and HPLC analysis was used to detect the degradation of OTA during different simulated digestion processes. The results of Fig.4 and Table 1 showed that OTA was slightly degraded *in vitro* digestive processes (degradation rate of 19.70%), and the degradation rates of OTA by S-CPA and M-CPA were 20.80% and 71.29% respectively. These results indicated that S-CPA was unstable in the simulated gastrointestinal environment, while M-CPA could tolerate gastrointestinal digestion and maintain a good OTA degradation ability.

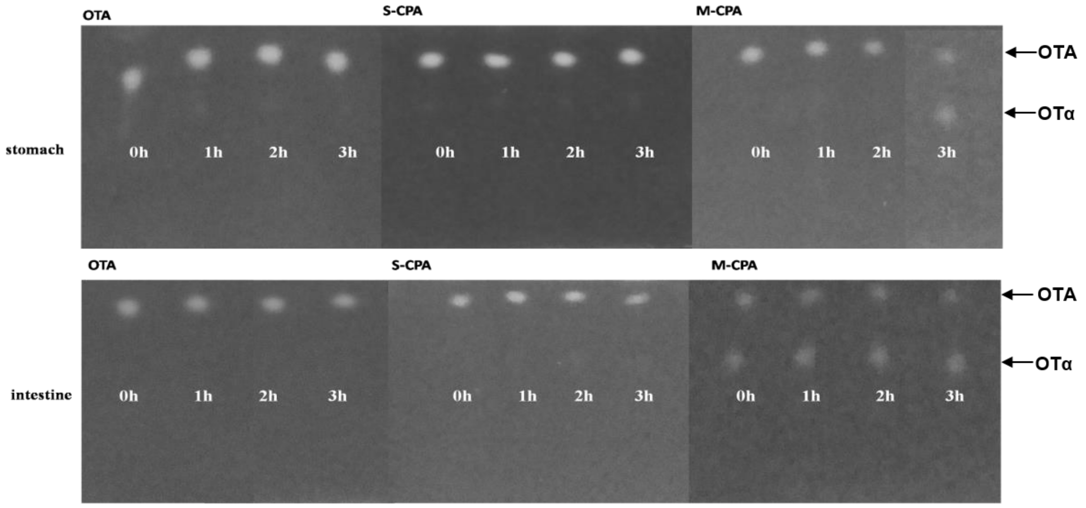

Figure S1. Thin layer chromatography of the recombinant M-CPA tested for OTA degradation during digestion in simulating gastrointestinal tract.

Table S1. Detoxification of OTA by M-CPA and S-CPA enzymes against control, during simulating gastrointestinal process.

Procedure	Digestion time (h)	CK	S-CPA	M-CPA
Stomach	0	0%	0%	0%
	1	9.98%	14.97%	21.08%
	2	8.24%	15.89%	44.37%
	3	15.08%	16.47%	57.51%
Small intestine	1	16.06%	17.44%	64.73%
	2	18.25%	18.41%	65.77%
	3	19.70%	20.80%	71.29%

2. Gene sequence and amino acid sequence of the CPA

2.1. GeneBank ID: NM_174750.2

>NM_174750.2 Bos taurus carboxypeptidase A1 (CPA1), mRNA
AGCTGACCTTCCCAACTGACTGCAGCATGCAGGGGCTGCTGATTTTGAGTGTGCTGCTGGG
GGCTGCCCTTGGCAAAGAGGACTTTGTGGGCCACCAGGTGCTCCGAATCACTGCCGCCGAT
GAGGCCGAGGTGCAGACGGTGAAGGAGCTGGAGGACCTGGAGCACCTGCAGTTGGACTTC
TGGAGGGGCCCTGGCCAGCCAGGCTCCCCCATCGACGTCCGAGTGCCCTTCCCCAGCCTCC
AGGCTGTTAAAGTCTTCCTGGAAGCCCATGGCATCAGATACAGGATCATGATCGAGGACGT
GCAGTCCCTGCTAGACGAGGAGCAGGAGCAGATGTTCGCCTCCCAGAGCCGGGCCCGCAG
CACCAACACATTTAACTACGCCACCTACCACACCCTGGATGAGATCTATGACTTCATGGAC
CTGCTGGTGGCCGAGCACCCACAGCTTGTCAGCAAACTCCAGATTGGCAGAAGCTATGAAG
GCCGTCCCATCTACGTGCTGAAGTTCAGCACTGGGGGAAGCAACCGTCCAGCCATCTGGAT
CGACTTAGGCATCCATTCCAGGGAGTGGATCACCCAGGCCACTGGGGTCTGGTTTGCAAAG
AAGTTCACAGAAGACTATGGCCAGGACCCGAGTTTCACCGCCATTCTTGACAGCATGGACA
TATTCTTGGAGATTGTCACCAACCCTGATGGTTTTGCCTTCACCCACAGCCAGAATCGATTG
TGGCGCAAGACTCGATCTGTCACGTCAAGCTCCCTCTGTGTTGGGGTGGACGCCAACCGGA
ACTGGGATGCCGGCTTTGGGAAGGCAGGAGCCAGCAGCAGCCCCTGCTCGGAGACTTATC
ATGGCAAGTATGCCAATTCTGAAGTGGAGGTCAAGTCCATCGTGGACTTTGTGAAAGACCA
TGGGAACTTCAAGGCCTTCCTCTCCATCCACAGCTACTCCCAGCTCCTCCTCTATCCCTATG
GCTACACAACACAATCAATCCCTGACAAGACTGAGCTGAATCAGGTGGCTAAGTCCGCTGT

TGAGGCCCTGAAGTCTCTGTATGGGACCAGCTACAAGTATGGCAGCATCATCACAACAATT
TACCAAGCCAGTGGAGGCAGCATTGACTGGTCCTACAACCAAGGCATCAAGTACTCCTTCA
CCTTTGAACTCCGGGACACGGGGCGCTATGGCTTCCTGCTGCCAGCCTCCCAGATCATCCCC
ACAGCCCAGGAGACGTGGCTGGGGGTTCTGACCATCATGGAGCACACGTTGAATAACCTCT
ACTGACCCAGCCCTCCAGCACTCTTTTCCTCCTCCTCTTCAGCCCTACCCAGGCAACCAAAT
AAAGTCTGAGAGTACATGG

2.2. GeneBank ID: CAA83955.1

>CAA83955.1 carboxypeptidase A [Bos taurus]
MQGLLILSVLLGAALGKEDFVGHQVLRITAADEAEVQTVKELEDLEHLQLDFWRGPGQPGSPID
VRVPFPSLQAVKVFLEAHGIRYRIMIEDVQSLLDEEQEQMFASQSRARSTNTFNYATYHTLDEIYD
FMDLLVAEHPQLVSKLQIGRSYEGRPIYVLKFSTGGSNRPAIWIDLGIHSREWITQATGVWFAKKF
TEDYGQDPSFTAILDSMDIFLEIVTNPDGFAFTHSQNRLWRKTRSVTSSSLCVGVDANRNWDAGF
GKAGASSSPCSETYHGKYANSEVEVKSIVDFVKDHGNFKAFLSIHSYSQLLLYPYGYTTQSIPDKTE
LNQVAKSAVAALKSLYGTSYKYGSIITTIYQASGGSIDWSYNQGIKYSFTFELRDTGRYGFLLPASQI
IPTAQETWLGVLTIMEHTVNNLY
